# Simultaneous Determination of Seven Active Components in Rat Plasma by UHPLC-MS/MS and Application to a Quantitative Study after Oral Administration of Huang-Lian Jie-Du Decoction in High Fat-Induced Atherosclerosis Rats

**DOI:** 10.1155/2019/5628160

**Published:** 2019-07-01

**Authors:** Li Jiang, Yanling Xiong, Lanbin Yu, Yu Chen, Qiyun Zhang, Xue Ding, Xiaojun Yan, Peng Nie, Guoliang Xu

**Affiliations:** ^1^Jiangxi Provincial Key Laboratory of TCM Etiopathogenesis, Jiangxi University of Traditional Chinese Medicine, Nanchang 330004, China; ^2^Key Laboratory of Pharmacology of Traditional Chinese Medicine in Jiangxi, Nanchang 330004, China; ^3^Pharmacy Department of Zhejiang Hospital, Hangzhou 310013, China; ^4^Center for Health System and Policy Research, Institute of Medical Information, Chinese Academy of Medical Sciences, Beijing 100020, China

## Abstract

Huang-Lian Jie-Du decoction (HLJDD) has been used to treat cardiovascular and cerebrovascular disease for many years in China. Currently, the determination of effect components in HLJDD is focusing either on the formula or on the extract, while quantification of that in biological samples is scarce, especially simultaneous determination of multicomponent. In this paper, a rapid, specific, and sensitive ultra-high performance liquid chromatography-tandem mass spectrometry method was developed and fully validated for the simultaneous determination of seven main active constituents, i.e., baicalin, baicalein, wogonoside, wogonin, berberine, palmatine, jatrorrhizine in rat plasma. The method was also successfully applied to a quantitative study after oral administration of HLJDD at different doses of 1.5, 3, and 6 g/kg body weight to high fat-induced atherosclerosis rats. The analytes were detected by ESI source and multiple reactions monitoring (MRM) using positive scanning mode. The blood was collected from the abdominal aorta of rats at predetermined time and preprepared with icariin and tetrahydropalmatine as internal standards (IS). Sample preparation was achieved by protein precipitation (PPT). The validation parameters (linearity, sensitivity, intra-/interday precision and accuracy, extraction recovery, and matrix effect) were within acceptable ranges, and biological extracts were stable during the entire storing and preparing process. And the result of determination of HLJDD-containing plasma, baicalin, baicalein, wogonoside, and wogonin could be highly detected in a dose-dependent manner while berberine, jatrorrhizine, and palmatine were determined in a very low level and in a dose-independent mode. Thus, the established method was sensitive enough and successfully applied to the determination of seven effective components in plasma taken from 24 high fat-induced atherosclerosis rats after oral administration of three dosages of HLJDD.

## 1. Introduction

Atherosclerosis (AS) is a chronic disease with a typical pathological process of abnormal lipid metabolism, i.e., infiltrating into the arterial intima and depositing on the vessel wall which triggers inflammation and then in turn leads to the abnormal response to injury of the vessel wall [1], such as alteration, exudation, and proliferation. These pathological changes are also prone to myocardial infarction, angina pectoris, arrhythmia, stroke, and severe sudden death. A large number of clinical and basic studies have shown that the risk factors for AS include hyperlipidemia, hypertension, hyperglycemia, obesity, and metabolic disorders of autologous bioactive substances [2]. Although the pathogenesis of AS is not fully understood, inflammation-injury-reaction theory proposed by Professor Ross [3] has been widely recognized around the world. The medicine used to treat AS currently is mostly anti-inflammatory drugs, and it has become a new way to prevent and treat AS. However, there is still a lack of effective drugs for AS. In TCM, there are many theories according to the pathogenesis of AS, such as damp heat (Shi Re in Chinese), toxic pathogen (Du Xie in Chinese), and phlegm stasis (Tan Yu in Chinese); that is to say, therapeutic principles of clearing away heat and dampness and detoxicating would be effective for AS.

**Figure 1:**
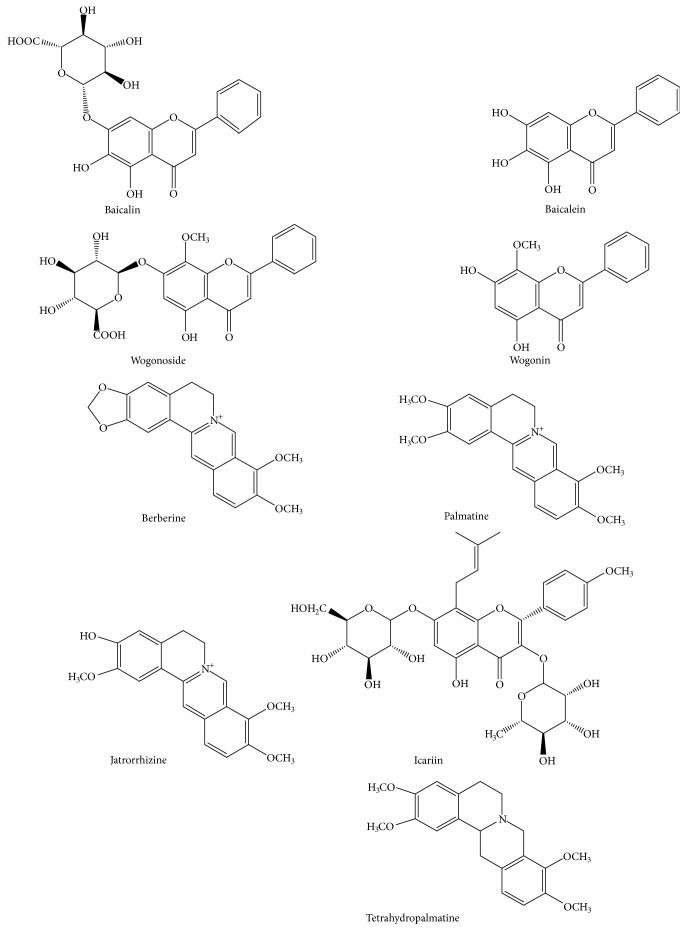
Chemical structures of baicalin, baicalein, wogonoside, wogonin, berberine, palmatine, jatrorrhizine, icariin (I.S.), and tetrahydropalmatine (I.S.).

Hang-Lian Jie-Du Decoction (HLJDD), a classic prescription of TCM, was composed of four common herbs at the ratio of 3:2:2:3:* Rhizome Coptidis*,* Radix Scutellariae*,* Cortex Phellodendri, *and* Fructus Gardeniae*. This formula, first recorded by a doctor named Wang Tao in his monograph “*Wai Tai Mi Yao*” (Chinese Tang Dynasty), had been used for 1700 years in China [4] and was mainly depending on clearing away heat-toxin to intervene in the inflammatory disorder, the basic pathophysiological process of AS [3]. The existing research showed that HLJDD has a number of pharmacological effects such as anti-inflammation, lowering blood sugar, regulating blood lipids, and antioxidation. Thus, it was used widely to treat diseases such as coronary heart disease [5], diabetes [6], hypertension [7], angina [8], and myocardial infarction [9] in clinic. Recently, accumulating evidence has shown HLJDD's efficacy on AS [10] and the components in HLJDD have been extensively studied in order to elucidate the mechanism. For example, berberine, the component of* Rhizome Coptidis* and* Cortex Phellodendri*, could inhibit the synthesis of lipids by activating AMP enzymes and controlling LDL receptor expression [11]. The extracts of* Radix Scutellariae* could significantly reduce the content of oxidation of LDL and suppress inflammatory responses in macrophages [12]. The effective ingredients in* Fructus Gardeniae* could reduce the fat by inhibiting the activity of pancreatic lipase [13]. In addition, alkaloids (e.g., berberine, palmatine, and jatrorrhizine), flavonoids (e.g., baicalin, baicalein, wogonoside, and wogonin), and iridoids (e.g., geniposide and shanzhiside) were the major active components of HLJDD according to the previous phytochemical research [14–16]. Thus, it was essential to quantify the above effective components accurately in HLJDD treated for AS. However, the reported pharmacokinetic studies of HLJDD failed to fully reflect the in vivo process of the compound due to its limited components in recent years. Moreover, to date, the multicomponents of this effective and traditional formula in biological samples (e.g., AS rats) have been nearly rarely provided so far. Generally, UHPLC-MS/MS has been regarded as one of the premier tools in complex biological samples, especially for properties of Chinese medicine formulae. Therefore, we developed a UHPLC-MS/MS method for simultaneous determination of the above components in rat plasma. Then the method was used to quantify the active ingredients (Figure 1) in AS rat plasma after three oral dosages of HLJDD.

## 2. Experimental

### 2.1. Materials and Chemicals

The medicinal materials of* Rhizome Coptidis*,* Radix Scutellariae*,* Cortex Phellodendri, *and* Fructus Gardeniae* were purchased from Jiangxi Jiangzhong Traditional Chinese Medicine Pieces Company and they were authenticated by Professor Shouwen Zhang (School of Pharmacy, Jiangxi University of Traditional Chinese Medicine, China). Baicalin was obtained from National Institutes for Food and Drug Control. Baicalein, wogonoside, wogonin, berberine, palmatine, jatrorrhizine geniposide, icariin, and tetrahydropalmatine were purchased from National Pharmaceutical Engineering Center for Solid Preparation in Chinese Herbal Medicine. The purity of these standards was more than 98.0%. HPLC-grade methyl alcohol (MeOH) and acetonitrile (ACN) were obtained from Merk (Merck, Germany), and formic acid and ammonium formate were of chromatographic purity and were purchased from DikmaPure (DikmaPure, USA) and Sigma (Sigma, USA), respectively. Deionized water was prepared by a Millipore Alpha-Q water purification system (Millipore, USA). Other chemicals and solvents were all of analytical grade.

### 2.2. Preparation of HLJJD

An amount of crude drug equivalent to a daily dose of Huang-Lian-Jie-Du decoction was weighed, and the four medicinal herbs (*Rhizome Coptidis, Radix Scutellariae, Cortex Phellodendri, *and* Fructus Gardeniae*) were mixed in the ratio 3: 2: 2: 3. The medicinal materials were soaked for 1 h and refluxed with water (1:6, w/v) for 30 min. Filtrates were collected and the residues were then refluxed in water (1:4, w/v) for 20 min. Two batches of filtrates were combined and the obtained solution was concentrated by a rotatory vacuum evaporator concentrated to 0.5g/mL crude herb. Then the extract was stored at 4°C before use.

### 2.3. Preparation of Stock and Working Solutions

Accurately weigh a certain amount of baicalin, baicalein, wogonoside, wogonin, berberine, palmatine, jatrorrhizine, icariin, and tetrahydropalmatine in a 25mL volumetric flask, dissolved and diluted to the concentration of 144, 92, 176, 140, 200, 116, 423, 480, and 488 *μ*g/mL, respectively, as the stock solutions. Then the stock solutions were further diluted into 21.0-26250, 20.0-17500, 20.0-18750, 20.0-1367, 0.50-781.25, 0.50-566.40, 0.50-250, 80.0, and 5.0 ng/mL, respectively, as the working solutions.

### 2.4. Preparation of Calibration Standard and Quality Control (QC) Samples

The calibration standard samples were prepared by freshly spiking the appropriate working solution into blank plasma yielding the concentrations of 2.1-2625, 2.0-1750, 2.0-1875, 2.0-136.72, 0.25-78.13, 0.25-56.64, and 0.25-25 ng/mL for baicalin, baicalein, wogonoside, wogonin, berberine, palmatine, and jatrorrhizine, respectively, and processed as described in the sample preparation. Quality control (QC) samples used for the intra- and interday accuracy and precision, extraction recovery, and stability study were prepared in the same way as calibration standard samples at concentrations of 2.1, 25.0, and 2625 ng/ml for baicalin; 2.0, 25.0, and 575 ng/ml for baicalein; 2.0, 25.0, and 1875 ng/ml for wogonoside; 2.0, 25.7, and 136.72 ng/ml for wogonin; and 0.25, 2.5, and 25.0 ng/ml for berberine, palmatine, and jatrorrhizine.

### 2.5. Plasma Sample Preparation

An aliquot of 100*μ*L thawed plasma sample was transferred into an Eppendorf tube (EP tube), to which 10 *μ*L of seven analytes and internal standards solution from each working solution were added. After being vortexed for 15 s, 100 *μ*L methanol plus 300 *μ*L acetonitrile was added and rested for 3 h following vortex mixing for 30 s to precipitate protein. Subsequently, the mixture was centrifuged at 15,000×g for 10 min. 400 *μ*L supernatant was transferred into another EP tube and evaporated to dryness under the stream of nitrogen in a water bath at 40°C. The residue was dissolved in 100 *μ*L of reconstituted solution which consisted of acetonitrile-0.1% formic acid (9:1, v/v) and then centrifuged at 18,000×g for 15 min after vortexing for 2 min. The supernatant was injected into the UHPLC–MS/MS system for analysis.

### 2.6. Instruments and Chromatographic Conditions

The analysis was performed using the Shimadzu UHPLC system (Shimadzu Corporation, Kyoto, Japan) consisting of an LC-30AD binary pump, a DGU-20A5 degassing unit, a SIL-30AC autosampler, and a CTO-30A5R column oven. Mass spectrometric detection was conducted on an AB Sciex Qtrap 5500 System (Applied Biosystems, Foster City, CA, USA) and equipped with Analyst software (version 1.6.2) for data processing. Chromatographic separation was achieved on a Shimadzu Shim-pack XR-ODS III (1.6 *μ*m, 2.0 mm×75 mm, Shimadzu Corporation, Kyoto, Japan). The mobile phase consisted of 5 mM ammonium formate-0.1% formic acid (solvent A) and acetonitrile (solvent B). The gradient elution conditions were optimized as follows: 10-20% B (0-3 min), 20-45% B (3-4.5 min), 45% B (4.5-7 min), 45-80% B(7-12 min), 80-95% B (12-14 min), 95-10%B (15-15.5 min), and 10% B (15.5-16 min). The flow rate was set at 0.3mL/min with the column temperature maintained at 40°C. The injection volume of 1 *μ*L was used for the reference standards and samples.

For mass detection, the electrospray ionization source was operated in positive mode. The operating parameters were optimized under the following conditions: 500°C for the interface temperature and 5.5 kV for the ion spray voltage; ion source gas 1 and gas 2 were fixed at 50 psi. Vacuum was obtained by a Turbo molecular pump (Agilent Technologies, USA). Nitrogen generated by the high purity nitrogen generator (99.999%, Peak Scientific Instruments Ltd., UK) was used as the source of curtain gas (30 psi) and collision gas (7 psi). The optimized multiple reaction monitoring (MRM) parameters including collision energy and declustering potential are listed in Table 1.

### 2.7. Validation of the UPLC–MS/MS Method

The proposed method was validated for specificity, extraction recovery, matrix effect, LLOQ, linearity, and stability. Meanwhile, intra-and interday validation were performed to evaluate the accuracy and precision of the measurements.

#### 2.7.1. Specificity, Linearity, Lower Limits of Detection, and Quantification

To investigate the specificity of this method, chromatogram comparison of blank plasma, blank plasma spiked with IS/analyte, and rat plasma samples were conducted to assay for the exclusion of any endogenous interference existing at or close to the expected retention time of the analytes. Calibration curves were established from peak area ratios (analyte peak area to the internal standard peak area, As/Ai) versus nominal concentrations using a linear least-squares regression model (1/X^2^ weighting).

#### 2.7.2. Precision and Accuracy

Intra- and interday precisions and accuracy were denoted by assessing measured results of QC samples at low, medium, and high concentrations. Each of the five samples was processed in parallel. Continuously measure a batch of samples and calculate intraday precision. Then, five samples of low, medium, and high plasma samples of each component were prepared in parallel on different consecutive days, and the 45 samples were tested in three batches, and the interday precision was calculated by referring to the accompanying standard. Precisions were expressed by the relative standard deviation (RSD, %), while accuracy (%) was presented as the percentage difference between the mean measured concentrations and the spiked concentrations.

#### 2.7.3. Extraction Recovery and Matrix Effect

Extraction recoveries of the seven analytes from rat blank plasma were determined by comparing the mean peak areas of the QC samples spiked before protein precipitation with those spiked after protein extraction. Matrix effects occurred when endogenous molecules were coeluting with the analytes of interest enhancing or decreasing the ionization efficiency of the electrospray interface. The matrix effect was assessed via comparing the mean peak areas of the QC samples spiked after the pretreatment with those of the pure solution.

#### 2.7.4. Stability

Stability of seven active constituents was checked by comparing measured results with those of freshly prepared samples. The short- and long-term stabilities were evaluated by analyzing QC plasma samples kept at room temperature for 4 h and in the freezer (−20°C) for 30 days, respectively; the freeze-thaw stability was carried out by detecting QC samples undergoing three freeze-thaw cycles; the postpreparation stability was assessed by determining the extracted QC samples stored under autosampler conditions (4°C) for 12 h.

### 2.8. Determination of HLJDD-Containing Plasma

Specifically pathogen-free Sprague-Dawley rats (male, weighing 200 ± 20 g) were purchased from Hunan Slac Laboratory Animal Co. LTD (Hunan, China, Certificate No. SCXK-2013-0004) and acclimated in Exhaust Ventilated Closed-System Cage Rack (EVC) for at least a week with environmentally controlled quarters (22±2°C and 12/12-h light/dark cycle) and free access to standard chow and water. Animal welfare and experimental procedures were strictly in accordance with the guide for the care and use of laboratory animal by the Animal Ethics Committee of Jiangxi University of TCM. After one week of acclimatization, the mice were randomly divided into five groups (*n*=12): normal control group, model group, and three dosage groups. The normal control group was fed with common diet, while the other groups were fed with high-fat diet (3% cholesterol, 0.5% sodium cholate, 0.2% propylthiouracil, 5% sugar, 10% lard, and 81.3% basic diet). The high fat-induced AS rat model was established in our previous study [17, 18]. Briefly, the AS model was made by the combination of regular intraperitoneal injection of vitamin D_3_ and high fat diet for 8 weeks. The model rat was injected with 600,000 U/Kg vitamin D_3_ on the second week and 200,000 U/Kg every other week. The three dosage groups were gavaged HLJDD at doses of 1.5, 3, and 6 g/ kg (low, medium, and high) once daily from the 3rd week for 8 weeks until they were sacrificed. Then, the heparinized blood samples were collected from abdominal aorta after 30 minutes of the last administration and frozen at −20°C until analysis.

## 3. Results

### 3.1. Method Validation

#### 3.1.1. Specificity

**Figure 2:**
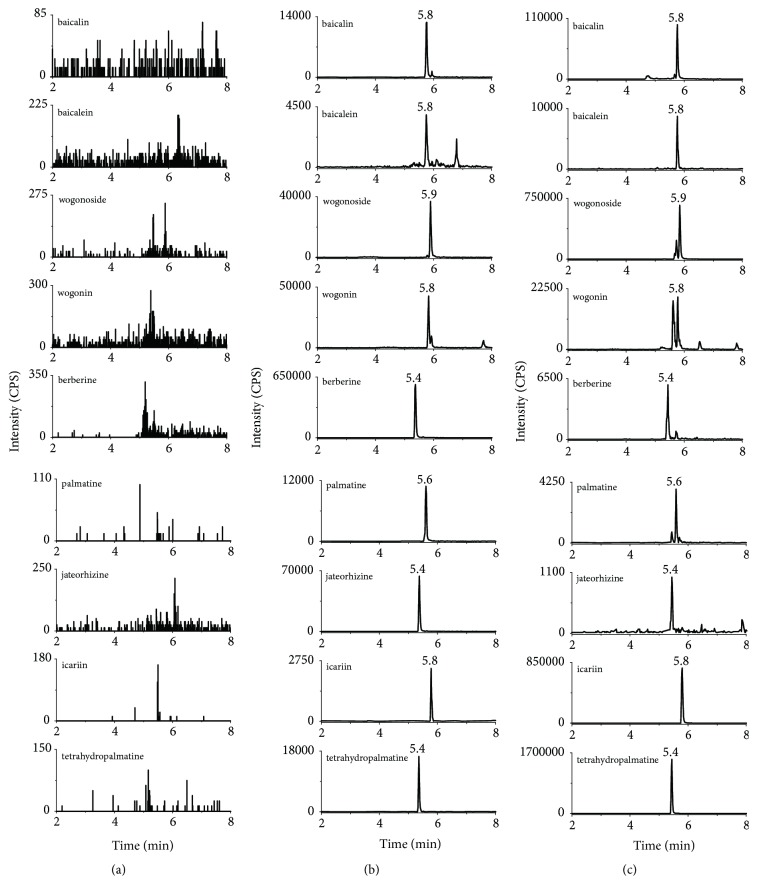
The MRM spectrum of each component (a) blank plasma; (b) blank plasma spiked with analytes and IS; (c) rat plasma sample collected after i.g. administration of HLJDD.

The total separation time for all analytes was 16 minutes and there were little interferential substances with the analytes and IS in the blank plasma. Representative chromatogram of analytes and IS in rat plasma was shown in Figure 2.

#### 3.1.2. Linearity

The calibration curve of each analyte was established with at least six points of standard solution, and each point was repeated five times. The calibration curves of all analytes exhibited good linearity, and the regression equations with correlation coefficients and linear range were listed in Table 2.

#### 3.1.3. Precision and Accuracy

The intraday and interday precision of all analytes were all less than 15%, whilst the accuracy deviation values were all within 96.4±6.0% of the actual values at each QC level (shown in Table 3).

#### 3.1.4. Extraction Recovery and Matrix Effect

The extraction recoveries (absolute recoveries) of each component were more than 80% at each QC level, which satisfied the quantitative requirements of biological samples. With respect to matrix effect, no suppressive or enhancing effect was found on the analytes and IS. That is to say, the responses of all components in the matrix were consistent with that in pure solution. The results are shown in Table 4.

#### 3.1.5. Stability

Results of the stability (shown in Table 5) illustrate that all analytes remained generally stable in plasma for 4 h when stored at room temperature or 30 days when stored at −20°C for three freeze-thaw cycles. And they showed satisfactory stability in the reconstituted solutions when stored under autosampler condition for 12 h.

#### 3.1.6. Application

**Figure 3:**
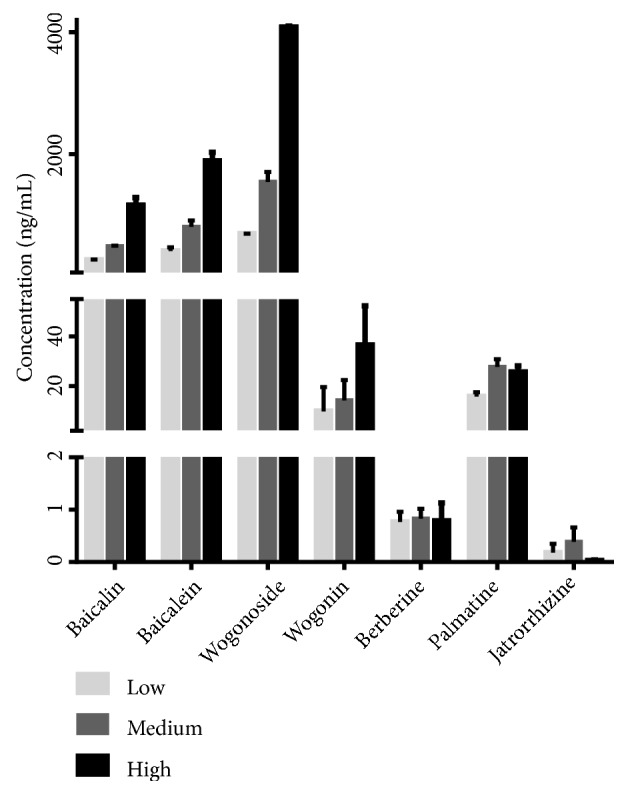
The concentration of seven analytes in rat plasma after oral administration of three dosages of HLJDD.

The present method was successfully used for the determination of three dosages of HLJDD in 24 high fat-induced AS rat plasmas. The concentration of the analytes in plasma after i.g. administration was shown in Table 6 and Figure 3.

## 4. Discussion

### 4.1. Optimization of LC–MS for Quantitative Analysis

The choice of mobile phase was a crucial factor in achieving fine chromatographic behavior and appropriate ionization. Modifiers such as formic acid and ammonium formate alone or in combination with different concentrations were compared. The best peak shape and ionization were achieved adapting 5 mM ammonium formate buffer. Linear gradient elution was used to elute endogenous substances residue from the column. In addition, all analytes and IS were both fully scanned by positive and negative mode. As alkaloids were detected overwhelmingly in the positive mode while flavonoids with little difference in both modes, the positive mode was used in the MRM acquisition.

### 4.2. Selection of the Determined Components

According to the previous phytochemical and HPLC–MS studies, iridoids, alkaloids, and flavonoids were the predominant constituents in HLJDD [19–23]. And many components of HLJDD could alleviate AS development by inhibiting the vascular inflammatory processes, which was the initiation and progression of AS [3, 4]. The flavonoids of* Radix Scutellariae*, i.e., baicalin, baicalein, and wogonin, could suppress vascular inflammation in vitro and in vivo [24], and wogonoside could modulate inflammatory mediator expression in LPS-induced RAW264.7 cells [25]. In addition, wogonin inhibited MMP-9 gene expression (a major role in the pathogenesis of AS) via MAPK signaling pathways [26]. Berberine, palmatine, and jatrorrhizine, the main alkaloids constituents in* Rhizome Coptidis* and* Cortex Phellodendri*, could suppress the formation and development of AS by altering gut microbiota compositions [27], anti-inflammation, and lowering blood lipids [28–30]. Thus, baicalin, baicalein, wogonoside, wogonin, berberine, palmatine, and jatrorrhizine were determined in high fat-induced AS rats.

Although the determination of HLJDD was reported before [31, 32], the active components of it in biological samples have seldom been reported. Deng and He [33, 34] determined baicalin and wogonoside in type 2 diabetic and normal rats. Zeng and Zhu [35, 36] quantified baicalin, geniposide, and berberine in MCAO rats. Nevertheless, they were all based on HPLC method and the LLOQ were *μ*g level for those constituents. Thus, we quantified the above seven ingredients simultaneously in the plasma of high fat-induced AS rats after oral administration of HLJDD except geniposide (for there was almost no geniposide in the drug-containing plasma, and the pretreatment of geniposide was extremely unstable) by UHPLC-MS/MS. In our study, the precision, accuracy, matrix effect, and stability under all conditions are within bioanalytical methodology validation acceptance criteria [37], with the extraction recovery of palmatine and berberine higher than Lu's study [15], the LLOQ was lower, and the retention time greatly shortened than the previous research [33–36]. Although some peaks eluted with closed retention time, the MS detector can determine them accurately by taking the advantage of its high selective MRM method.

### 4.3. Concentration Profiles of the Analytes in AS Plasma

From the result of determination of HLJDD-containing plasma, baicalin, baicalein, wogonoside, and wogonin could be highly detected in a dose-dependent manner while berberine, jatrorrhizine, and palmatine were determined in a very low level and in a dose-independent mode. For one thing, the former absorptions were relatively better than the latter. For another, flavonoids are easily bound to glucuronic acid or sulfuric acid to form two-phase metabolism so their plasma concentration-time curves showed obvious bimodal phenomena and concentration increased slowly from the 5th day [38]. However, even long-term administration of berberine and other alkaloids was not easy to accumulate in vivo for their poor absorption through the gut wall. First of all, berberine had strong rigidity and poor solubility for it is a quaternary ammonium alkaloid with conjugated double bonds. Besides, berberine was the substrate of P-gp, which is an efflux transporter. All these factors lead to the poor absorption of berberine. Secondly, most of them were excluded by the gastrointestinal tract after intragastric administration of berberine and they were also metabolized through various other pathways at the same time [39, 40]. Moreover, the distribution of berberine in the organs was much higher than that in the blood, such as liver, kidney, and muscle [41]. Pharmacokinetic studies [15, 39] indicated the blood clearance of berberine was very fast and its biotransformation in the liver was rapid and substantial. So the first pass elimination of intestinal tract and the tendency distribution of liver could also lead to the low concentration of berberine in blood. Furthermore, our previous studies [42] have shown that baicalin is a partial agonist of berberine, which weakened the pharmacological effect of berberine in a higher concentration range. Therefore, it may contribute to the low blood concentration of berberine in vivo. Likewise, the structures of jatrorrhizine and palmatine are similar to that of berberine, so the low blood concentration of these two alkaloids may also be caused by similar reasons with berberine for the principle of structural similarity.

As we all know, the absorption of the intestinal tract and metabolism of the liver may affect the bioavailability of the drug [43] and the pathological conditions; i.e., AS may also affect the process of the drug in the body. In humans, the development of metabolic diseases including AS has closely related to imbalance intestinal flora [44] and the intestinal flora may also affect the process of drugs in vivo. So the effect of AS on the above constituents cannot be ignored.

## 5. Conclusions

Quantification of ingredients at a low level was the obstacles in the study of active components of traditional Chinese medicine in biological fluids. Simply using chromatography was usually time-consuming, insensitive, and nonselective enough. In the present study, a highly selective and sensitive UHPLC–ESI-MS method was developed and validated to simultaneously determine the seven active components in rat plasma and successfully applied to 24 high fat-induced AS rats after oral administration of HLJDD. It could apply for further pharmacokinetic study of the analytes and may provide a scientific basis for clinical application of HLJDD.

## Figures and Tables

**Table 1:** Optimized precursor/production pairs and multiple reaction monitoring (MRM) parameters for the analytes and IS.

Analyte	m/z	DP/V	CE/eV
Baicalin	447.1→271.0	95	30
Baicalein	271.0→123.0	190	44
Wogonoside	461.1→285.0	90	28
Wogonin	285.0→270.0	130	34
Berberine	337.8→294.0	80	38
Palmatine	353.9→322.1	50	54
Jatrorrhizine	339.1→295.0	100	27
Icariin(IS)	677.3→369.1	200	70
Tetrahydropalmatine(IS)	357.2→192.1	210	35

**Table 2:** Regression data and LLOQs of the multi-components determined in HLJDD.

Analyte	Linear range (ng/mL)	Linear regression equations	Correlation coefficient (*r*)	LLOQs (ng/mL)
Baicalin	2.1-2625	y =35.46x+1561	0.9995	2.1
Baicalein	2.0-1750	y=2.522x+233.2	0.9989	2.0
Wogonoside	2.0-1875	y=669.6.x+55174	0.9949	2.0
Wogonin	2.0-136.7	y=5885x+30206	0.9985	2.0
Berberine	0.25-78.13	y=28947x+29485	0.9989	0.25
Palmatine	0.25-56.64	y=4786x+1293	0.9955	0.25
Jatrorrhizine	0.25-25.0	y =2877x+2720	0.9975	0.25

**Table 3:** Intra-/inter-day precision and accuracy for the determination of the components in rat plasma.

Analytes	Spiked Concentration (ng/mL)	Precision (%)		Accuracy (%, Mean ± SD)	
		Intra-day	Inter-day	Intra-day	Inter-day
Baicalin	2.1	6.54	2.96	85.12 ± 5.82	90.10 ± 9.84
	25.0	5.82	10.87	90.73 ± 7.80	87.45 ± 4.95
	2625	10.76	11.71	91.19 ± 8.37	94.14 ± 7.30
Baicalein	2.0	6.60	5.41	95.19 ± 8.95	92.61 ± 7.28
	25.0	11.72	7.98	96.46 ± 8.17	98.19 ± 6.89
	575	6.86	4.24	100.15 ± 9.36	105.85 ± 7.94
Wogonoside	2.0	3.45	14.31	87.12 ± 5.84	102.19 ± 9.78
	25.0	7.85	7.54	90.73 ± 7.91	85.96 ± 5.85
	1875	7.19	0.77	101.14 ± 5.38	98.89 ± 7.23
Wogonin	2.0	2.91	5.97	96.45 ± 5.89	88.77 ± 4.93
	25.7	4.53	14.11	92.76 ± 6.89	94.75 ± 5.73
	136.7	2.06	9.92	95.71 ± 8.78	90.50 ± 3.35
Berberine	0.25	3.92	13.28	88.19 ± 6.59	88.14 ± 5.05
	2.50	9.48	2.99	99.45 ± 8.84	85.79 ± 8.78
	25.0	1.29	12.25	100.89 ± 8.73	91.16 ± 4.38
Palmatine	0.25	3.75	10.54	85.12 ± 5.81	95.19 ± 8.95
	2.50	7.36	9.66	90.89 ± 7.93	106.49 ± 8.19
	25.0	6.28	0.78	108.78 ± 4.73	110.14 ± 9.24
Jatrorrhizine	0.25	3.55	13.06	104.12 ± 7.46	87.02 ± 6.24
	2.50	7.18	6.57	92.57 ± 6.65	90.64 ± 5.93
	25.0	4.38	0.86	98.10 ± 4.35	96.18 ± 4.97

**Table 4:** Extraction recovery and matrix effect of the components in rat plasma.

Analyte	Spiked Concentration (ng/mL)	Extraction recovery (%)	Matrix effect (%)
Baicalin	2.1	83.78 ± 7.53	106.60 ± 4.96
	25.0	93.53 ± 9.84	104.82 ± 9.12
	2625	93.45 ± 7.15	95.12 ± 4.89
Baicalein	2.0	82.18 ± 5.85	96.88 ± 5.45
	25.0	89.45 ± 4.56	91.18 ± 14.56
	575	103.29 ± 8.59	93.13 ± 4.89
Wogonoside	2.0	89.78 ± 6.66	102.51 ± 11.29
	25.0	89.40 ± 5.56	103.75 ± 13.05
	1875	96.75 ± 12.82	101.02 ± 11.82
Wogonin	2.0	92.51 ± 4.12	96.14 ± 6.85
	25.7	80.52 ± 9.20	98.16 ± 10.85
	136.7	89.66 ± 12.13	99.02 ± 13.63
Berberine	0.25	95.35 ± 12.51	91.97 ± 9.50
	2.50	96.26 ± 14.99	105.17 ± 8.57
	25.0	91.89 ± 12.23	107.46 ± 5.36
Palmatine	0.25	91.06 ± 10.29	92.25 ± 7.42
	2.50	92.17 ± 6.98	107.22 ± 11.41
	25.0	89.80 ± 8.45	87.49 ± 9.29
Jatrorrhizine	0.25	88.91 ± 13.32	90.24 ± 9.03
	2.50	93.33 ± 12.98	98.79 ± 8.57
	25.0	87.78 ± 4.89	110.67 ± 10.97

**Table 5:** Stability of the components in rat plasma under a variety of storage and process conditions.

Analyte	Spiked Concentration (ng/mL)	RSD%			
		Freeze-thaw cycles	Short-term stability	Long-term stability	Auto-sampler stability
		(three freeze-thaw cycles)	(room temperature, 4 h)	(-20°C, 30 d)	(4°C, 12 h)
Baicalin	2.1	13.86	10.53	5.63	6.93
	25.0	5.34	4.56	5.91	8.72
	2625	10.64	8.45	1.25	2.16
Baicalein	2.0	8.17	6.48	3.99	12.79
	25.0	7.89	5.78	6.23	7.89
	575	3.73	6.78	5.69	11.36
Wogonoside	2.0	5.54	3.91	2.46	7.88
	25.0	10.35	4.99	14.16	2.52
	1875	11.95	11.56	11.99	8.38
Wogonin	2.0	4.25	8.72	1.96	4.53
	25.7	7.11	12.12	3.79	3.73
	136.7	13.62	10.56	2.88	4.79
Berberine	0.25	12.30	7.63	3.73	7.82
	2.50	11.01	8.43	12.19	10.13
	25.0	14.52	4.96	13.13	7.40
Palmatine	0.25	15.75	8.45	13.83	9.93
	2.50	11.82	8.31	11.98	6.67
	25.0	10.07	4.97	2.54	7.99
Jatrorrhizine	0.25	11.20	7.54	2.86	7.87
	2.50	11.16	6.08	2.11	11.69
	25.0	14.82	9.05	1.31	4.09

**Table 6:** The concentration of seven analytes in rat plasma after oral administration of three dosages of HLJDD.

Analyte	Groups	Concentration (ng/mL)
Baicalin	L	262.95 ± 4.75
	M	489.06 ± 7.29
	H	1159.32 ± 126.39

Baicalein	L	406.48 ± 62.62
	M	804.98 ± 104.02
	H	1885.93 ± 143.94

Wogonoside	L	690.56 ± 4.33
	M	1542.70 ± 165.74
	H	4079.52 ± 21.00

Wogonin	L	9.89 ± 9.76
	M	14.21 ± 8.17
	H	36.50 ± 15.72

Berberine	L	0.77 ± 0.19
	M	0.83 ± 0.19
	H	0.79 ± 0.34

Palmatine	L	15.75 ± 1.82
	M	27.73 ± 3.10
	H	25.64 ± 2.49

Jatrorrhizine	L	0.18 ± 0.17
	M	0.39 ± 0.27
	H	0.03 ± 0.01

## Data Availability

The data used to support the findings of this study are available from the corresponding author upon request.
